# ZnO Nanorods Grown on Rhombic ZnO Microrods for Enhanced Photocatalytic Activity

**DOI:** 10.3390/nano12173085

**Published:** 2022-09-05

**Authors:** Yufu Zhu, Jiaying Yan, Lei Zhou, Liangdong Feng

**Affiliations:** 1 Jiangsu Provincial Engineering Research Center for Biomedical Materials and Advanced Medical Devices, Faculty of Mechanical & Material Engineering, Huaiyin Institute of Technology, Huaian 223003, China; 2 Faculty of Mathematics and Physics, Huaiyin Institute of Technology, Huaian 223003, China; 3 Department of Chemical Engineering, Huaiyin Institute of Technology, Huaian 223003, China

**Keywords:** hierarchical ZnO micro/nanostructures, photocatalytic activity, rhombic ZnO microrods

## Abstract

In this paper, the formation of rhombic ZnO microrods surrounded by ZnO nanorods was realized on the surfaces of zinc foils using a hydrothermal method. The photocatalytic degradation of Rhodamine B solution was used to test the photocatalytic performance of the prepared samples. Compared with the rhombic Zn(OH)F and ZnO microrods grown on zinc foils, the hierarchical micro/nanostructures formed by ZnO nanorods surrounding the surfaces of rhombic ZnO microrods have better photocatalytic performance. The experimental results are mainly due to the fact that the hierarchical ZnO micro/nanostructures formed by ZnO nanorods surrounding the surface of the rhombic ZnO microrods have a larger surface area compared with the rhombic Zn(OH)F and ZnO microrods. More importantly, the photocatalytic circulation experiments indicate that ZnO nanorods grown on rhombic ZnO microrods can be recycled and have a relatively stable photocatalytic performance.

## 1. Introduction

With the increasingly serious pollution problem, a variety of methods were used to solve the problem of environmental pollution [1,2,3,4,5,6,7,8]. Among the methods used for solving the environmental pollution problem, photocatalytic technology has attracted much attention because of the fact that clear and renewable solar energy can be used to degrade pollutants [3,4,5,6,7,8]. Thus far, a variety of semiconductor materials, such as TiO_2_, ZnO, WO_3_, CuO, CeO_2_, and their composites, have been widely studied for photocatalysis applications [9,10,11,12,13,14,15,16,17,18,19,20,21,22,23,24,25,26,27,28]. Among these materials, ZnO has been widely studied because it is non-toxic and easy to synthesize [18,19,20,21,22,23,24,25,26,27,28]. Up to now, powdery ZnO in various morphologies, including nanowires, spherical structures, and hierarchical structures, have been successfully prepared and used to study their photocatalytic properties [18,19,20,21,22,23,24,25,26,27,28].

At present, those micro- and nanostructures used for photocatalytic applications are mainly in powdery form. The problem with powdery materials is that they are difficult to separate after photocatalysis, so the powdery photocatalytic materials are difficult to be reused. This problem can be solved by preparing ZnO micro/nanostructures on the surfaces of substrates. Silicon, glass, aluminum foil, and nickel foam have been used for ZnO growth [29,30,31,32]. The obtained samples have been successfully applied in photocatalysis. ZnO prepared on the surfaces of those substrates is usually a single structure of nanowire or nanorod, which has been studied widely [31,32]. However, the fabrication of hierarchical ZnO micro/nanostructures on the substrate surface for photocatalysis applications has rarely been reported.

In this paper, the synthesis of Zn(OH)F on the surface of zinc foils was first realized, and then the preparation of hierarchical ZnO micro/nanostructures is realized by the growth of ZnO nanorods from the ZnO seed introduced on the surface of rhombic ZnO microrods, which is formed by calcinating Zn(OH)F microrods. The prepared ZnO nanorods grown on rhombic ZnO microrods have been successfully applied to photocatalysis. The photocatalytic performance test results show that the hierarchical ZnO micro/nanostructures grown on the surfaces of zinc foils have better photocatalytic performance than the Zn(OH)F and ZnO microrods grown on the surfaces of zinc foils. The cyclic experiment shows that the photocatalytic performance of hierarchical ZnO micro/nanostructures decreases slightly through three cyclic experiments.

## 2. Materials and Methods

### 2.1. Materials

All the chemical reagents used for the fabrication of the ZnO seed layer, Zn(OH)F microstructures, and ZnO nanorods are of analytical grade. Ammonium fluoride was purchased from Sinopharm Chemical Reagent Co., Ltd. (Shanghai, China). Zinc acetate, ethanolamine, zinc nitrate hexahydrate, and hexamethylenetetramine (HMT) were bought from Sigma-Aldrich (Steinheim, Germany). No further purification was carried out for all the chemical reagents during the experimental process.

### 2.2. Preparation of Zn(OH)F Microstructures

The zinc foils were cleaned with deionized water and ethanol alternatively. The above process was repeated three times. The cleaned zinc foils were transferred in a 300 mL solution. The composition of the solution is 0.025 M zinc nitrate hexahydrate and 0.05 M ammonium fluoride. The hydrothermal process was carried out at 120 °C for 4 h.

### 2.3. ZnO Nanorods Grown on Rhombic ZnO Microrods

In order to convert the obtained Zn(OH)F microstructures to ZnO ones and simultaneously form a layer of ZnO seed layer on the surface of the synthesized microstructures, the obtained sample in the first step was immersed in an ethanol solution consisting of zinc acetate and ethanolamine. After being totally dried, the sample was calcinated at 350 °C for 1.5 h.

After the samples were cooled naturally to room temperature, they were transferred into a solution containing 25 mM zinc nitrate and 25 mM HMT at 92 °C for 3 h. Before characterization, the samples were washed with deionized water, dried at room temperature and finally calcinated at 350 °C for 1.5 h.

### 2.4. Characterization

The morphologies of the samples obtained during the experimental process were observed by a field emission scanning electron microscope (FESEM; Quanta 250 FEG, Thermo Fisher Scientific, Waltham, MA, USA). The compositions of the samples were confirmed by an energy-dispersive X-ray spectrometer (EDX; INCAX-Max 20; Oxford, UK). X-ray diffraction (XRD) measurements were carried out on a Bruker AXS D8 ADVANCE X-ray diffractometer, Billerica, Germany.

### 2.5. Photocatalytic Activity Tests

The photocatalytic decolorization of the rhodamine B (RhB) solution was used to evaluate the photocatalytic activities of the samples. The absorbance of the photodegraded RhB solution was recorded on a Shimadzu UV-3600 UV-Vis spectrophotometer. During the experimental process, rod-like Zn(OH)F, rod-like ZnO, and hierarchical ZnO micro/nanostructures grown on zinc foils with a diameter of 8 cm were placed at the bottom of a photoreactor and immersed in 80 mL RhB solution with a concentration of 10 mg/L. The samples immersed in RhB solution were first kept in the dark environment for 30 min to establish the adsorption–desorption equilibrium, and then the RhB solution was placed under simulated light. The distance between the light source and sample is 16 cm. The simulated light source used in the experiment was provided by a PLS-SXE300C Xenon lamp. In recycling experiments, the used sample was washed with deionized water, dried at room temperature, and reused two additional times to assess the reusability of the catalyst.

## 3. Results and Discussion

**Figure 1:**
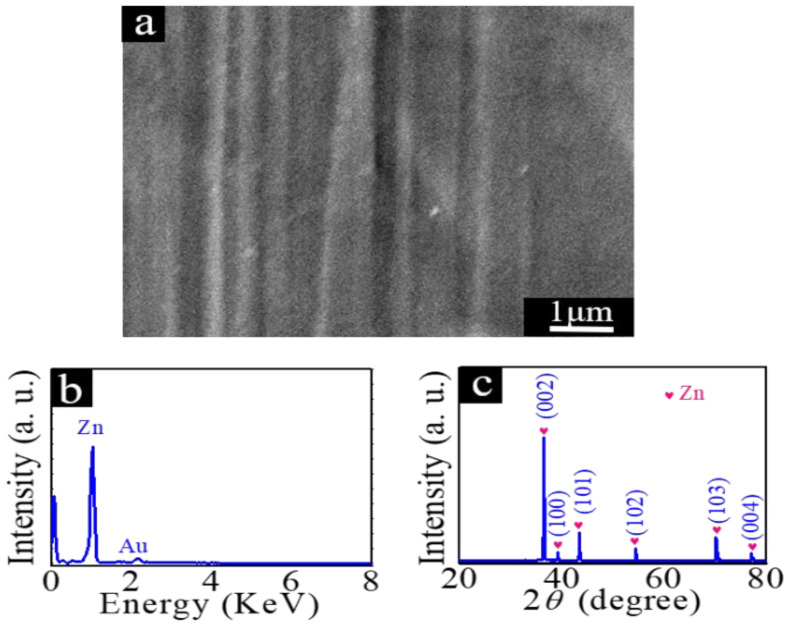
(**a**) SEM image of zinc foil, (**b**) EDX spectrum, and (**c**) XRD diffraction patterns of zinc foil.

The surface morphology of the zinc foil cleaned with deionized water and ethanol is shown in Figure 1a, from which it can be seen that the surface of the zinc foil is clean. The EDX result displayed in Figure 1b further indicates that the zinc foils used in this experiment are very pure. The Au signal observed in the spectrum comes from the gold film sputtered on the sample surface for SEM characterization. The XRD spectrum in Figure 1c indicates that the diffraction peaks agree well with the hexagonal phase of Zn (Joint Committee for Powder Diffraction Standards (JCPDS) No. 04-0831) [33,34].

**Figure 2:**
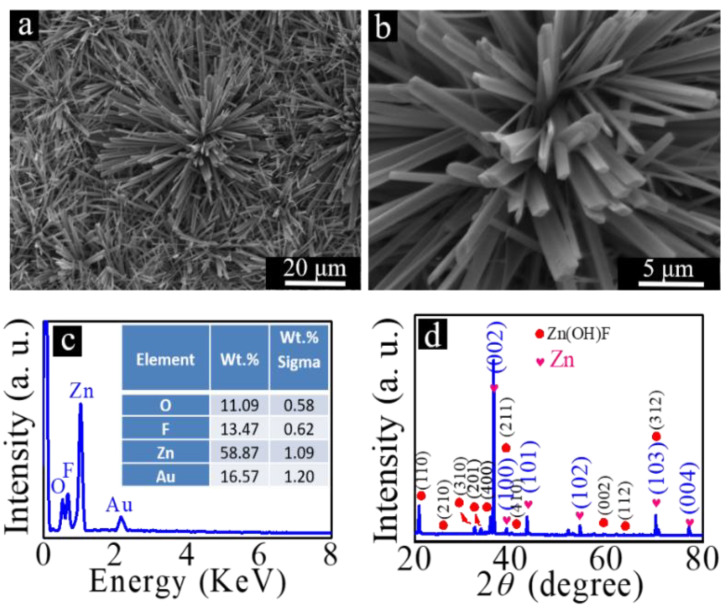
(**a**) Low- and (**b**) high-magnification SEM images of Zn(OH)F synthesized on zinc foil, (**c**) EDX spectrum, and (**d**) XRD diffraction patterns of zinc foil with Zn(OH)F grown on its surface.

After transferring the cleaned zinc foils into the prepared chemical solution, the solution was then sealed in an autoclave equipped with a Teflon liner, and finally, the autoclave was placed in a drying oven, and the autoclave was heated at 120 °C for 4 h. Rod-like Zn(OH)F was prepared on the surface of zinc foil through the hydrothermal reaction. The results are shown in Figure 2a,b. From the high-magnification SEM image shown in Figure 2b, it can be seen that rhombus-shaped cross-sectional Zn(OH)F microrods were synthesized. We believe that the growth mechanism of Zn(OH)F microrods can be described as follows: Zn(OH)F nanoparticles generated by the hydrothermal reaction in the solution first heterogeneously nucleated onto the surface of zinc foil, and then the rhombus-shaped Zn(OH)F grows from the crystal nucleus to form rod-like Zn(OH)F, as shown in Figure 2b [35].

After rod-like Zn(OH)F was grown on the surface of zinc foil, the composition of the obtained samples was analyzed. The EDX spectrum is shown in Figure 2c. Compared with the EDX spectrum in Figure 1b corresponding to the zinc foil, the new peaks of F and O elements appearing in this spectrum should come from the growth of rod-like Zn(OH)F on the surface of the zinc foil. From Figure 2c, it can be seen that the weight percentages of Zn, O, and F are 58.87%, 11.09%, and 13.47%, respectively. The calculated atomic ratio of O to F is about 1. To further investigate the crystal structure of the prepared samples, XRD analysis was carried out, and the results are shown in Figure 2d. Compared with the XRD spectrum demonstrated in Figure 1c, some new diffraction peaks appear. Except for those diffraction peaks from zinc foil, the newly appeared diffraction peaks arise from orthorhombic Zn(OH)F (JCPDS No. 32-1469). No other diffraction peak appears; we believe that the product prepared on the surface of zinc foil is orthorhombic Zn(OH)F.

**Figure 3:**
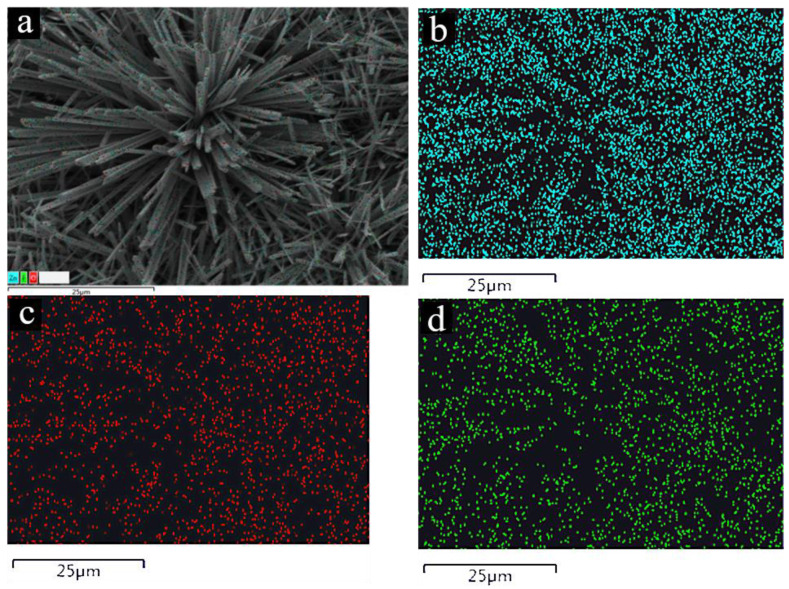
SEM-EDX elemental mapping images: (**a**) the elemental distribution image of zinc foil with Zn(OH)F grown on its surface; the elemental distribution data of (**b**) Zn, (**c**) O, and (**d**) F elements.

In order to further analyze the composition of the prepared products, EDX element mapping was used to analyze the samples. The results are shown in Figure 3a–d. From the EDX elemental mappings of Zn, O, and F, which are respectively demonstrated in Figure 3b–d, one can clearly see the distribution of elements.

**Figure 4:**
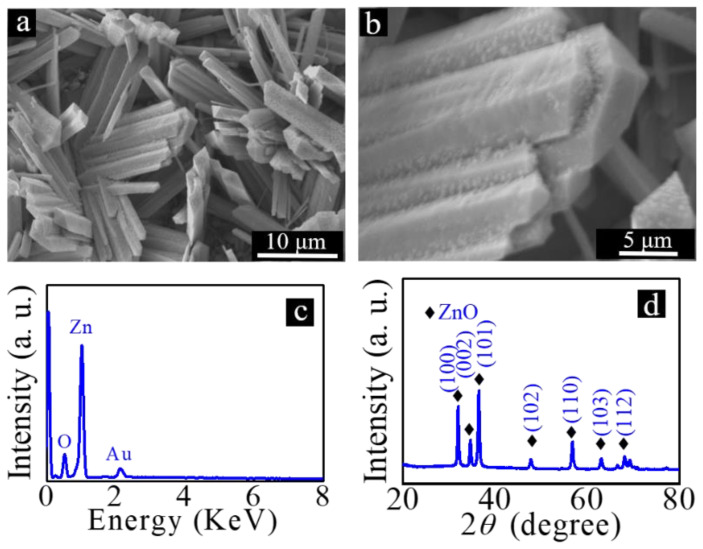
(**a**) Low- and (**b**) high-magnification SEM images of rod-like ZnO with ZnO seed layer loaded on its surface; (**c**) EDX spectrum and (**d**) XRD diffraction patterns of rod-like ZnO.

In order to realize the preparation of hierarchical micro/nanostructures, a ZnO seed layer was introduced onto the surface of the prepared rod-like microstructures. The samples corresponding to that shown in Figure 2a were first dipped into an ethanol solution prepared with zinc acetate and ethanolamine. The sample was then taken out and totally dried. Finally, the obtained sample was calcinated at 350 °C for 1.5 h. The morphology of the yielded sample is shown in Figure 4a. Compared with Figure 2a, no significant morphology changes were observed. From the high-magnification SEM image presented in Figure 4b, it can be clearly seen that some nanoparticles appear on the surfaces of the rod-like microstructures. We believe that these newly formed nanoparticles are ZnO. The introduction of these nanoparticles provides the necessary conditions for the subsequent preparation of ZnO nanorods on the surface of the rod-like microstructure. The composition and crystal structures of the calcined samples were analyzed. The results are shown in Figure 4c,d. From Figure 4c, it can be seen that the peak corresponding to element F disappears. We believe that the rod-like Zn(OH)F has been decomposed into ZnO. From the XRD results demonstrated in Figure 4d, it can be seen that all the diffraction peaks are consistent with those of hexagonal wurtzite ZnO (JCPDS No. 36-1451), and there are no other impurity peaks. These results further prove that rod-like Zn(OH)F is decomposed into ZnO by calcination, and ZnO nanoparticles are introduced on the surface of the obtained rod-like ZnO.

**Figure 5:**
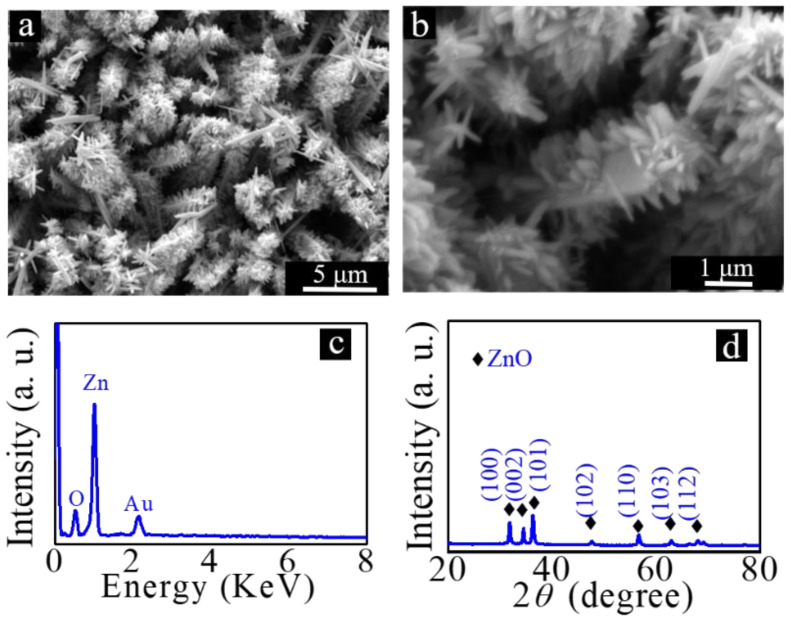
(**a**) Low- and (**b**) high-magnification SEM images of hierarchical ZnO micro/nanostructures; (**c**) EDX spectrum and (**d**) XRD diffraction patterns of hierarchical ZnO micro/nanostructures.

The introduction of ZnO nanoparticles on the surface of rod-like ZnO makes it possible to prepare hierarchical ZnO micro/nanostructures. Transferring zinc foils with rod-like ZnO on their surfaces into a solution containing 25 mM zinc nitrate and 25 mM HMT, the solution was then sealed in an autoclave equipped with a Teflon liner, and finally, the autoclave was placed in a drying oven, and the autoclave was heated at 92 °C for 3 h. After the hydrothermal reaction, the hierarchical ZnO micro/nanostructures can be prepared. The results are shown in Figure 5a,b. From the high-magnification SEM image demonstrated in Figure 5b, it can be seen that ZnO nanorods were grown on the surfaces of rod-like ZnO, and the synthesis of hierarchical ZnO micro/nanostructures was realized. The composition and crystal structures of the prepared hierarchical ZnO micro/nanostructures were analyzed. The EDX results are shown in Figure 5c. From the results, it can be seen that no other impurity elements can be observed. The XRD results shown in Figure 5d further prove that the prepared hierarchical ZnO micro/nanostructure is hexagonal wurtzite ZnO.

**Figure 6:**
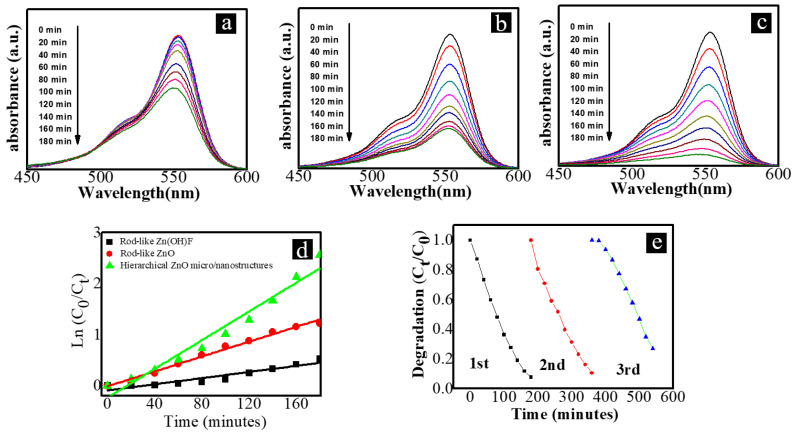
The absorbance of the solutions obtained by the degradation of the RhB solution with (**a**) rod-like Zn(OH)F, (**b**) rod-like ZnO, and (**c**) hierarchical ZnO micro/nanostructures. (**d**) The kinetic curves corresponding to the photodegradation of RhB with different micro/nanostructures. (**e**) The three cycling runs of the photodegradation ability of the hierarchical ZnO micro/nanostructures.

In order to study the photocatalytic performance of the prepared samples, the prepared rod-like Zn(OH)F, rod-like ZnO with ZnO seed layers on their surfaces, and hierarchical ZnO micro/nanostructures were used to test the photocatalytic performance. The photodegradation of the RhB solution was applied to evaluate the photocatalytic activity of the prepared products. During the experimental process, a small amount of the RhB solution was taken out of the degradation solution every 20 min to test the absorbance of the solution. Figure 6a–c, respectively, shows the absorbance of the solutions obtained by degradation of the RhB solution with zinc foils with rod-like Zn(OH)F, rod-like ZnO, and hierarchical ZnO micro/nanostructures on their surfaces at different time intervals. From the comparison of the results demonstrated in Figure 6a,b, it can be seen that the photodegradation rate of RhB with rod-like Zn(OH)F is lower than that with rod-like ZnO.

The photodegradation experiment can be divided into two stages [36]. In the first stage, the sample was immersed in rhodamine B solution, and the solution was kept in a dark environment. During this process, a small part of the dye will be adsorbed by the catalyst surface. This process is physical adsorption and does not involve photodegradation. In the second stage, under the irradiation of the simulated light source, the catalyst will absorb photon energy to generate electron-hole pairs, and the photogenerated electrons will interact with adsorbed O_2_ molecules to form ^•^O_2_^−^. Moreover, the holes can react with OH^−^ or H_2_O to form active ^•^OH radicals [37,38]. The produced ^•^O_2_^−^ and ^•^OH radicals can degrade dye molecules for the formation of H_2_O, CO_2_, and less dangerous products [39].

As we all know, the photocatalytic performance of a catalyst is related to the dimension, morphology, and surface area of micro/nanostructures [40]. Previous studies have shown that the band gap of Zn(OH)F and ZnO is about 3.2 eV [41,42]. Consequently, the band gap of Zn(OH)F before and after heat treatment does not change too much [41,42]. In addition, compared with the rhombic Zn(OH)F microrods shown in Figure 2b, the morphology of ZnO microrods demonstrated in Figure 4a does not change significantly. Therefore, the band gap and morphology of Zn(OH)F microrods before and after heat treatment do not change much, which should not be the main reason for the improvement of photocatalytic activity. Yang et al. have reported that Zn(OH)F has photocatalytic activity, and its photocatalytic ability is affected by heat treatment temperature [41]. When Zn(OH)F is thermally decomposed into ZnO, the photocatalytic performance of the obtained micro/nanostructure is improved [40]. Thus, we believe the increase in photocatalytic activity may be due to the structural changes induced by calcination [40].

By comparing the results demonstrated in Figure 6b,c, it can be seen that the photocatalytic performance of ZnO micro/nanostructures is improved after the growth of ZnO nanorods on the surface of rhombic ZnO microrods. We believe that the growth of ZnO nanorods on the surfaces of rhombic ZnO microrods can increase the amount of ZnO loaded on the surface of zinc foil. The surface area of hierarchical ZnO micro/nanostructures is the sum of the surface area of rhombic ZnO microrods and the surface area of ZnO nanorods. Therefore, compared with the rhombic ZnO microrods shown in Figure 4a, the surface area of hierarchical ZnO micro/nanostructures demonstrated in Figure 5a is increased. Therefore, the photocatalytic performance of the prepared hierarchical ZnO micro/nanostructure is improved. This study provides a new way to improve the photocatalytic performance of ZnO micro/nanostructures.

In order to further demonstrate the degradation ability of the obtained micro/nanostructures (rod-like Zn(OH)F, rod-like ZnO, and hierarchical ZnO micro/nanostructures), the kinetic curves corresponding to the photodegradation of RhB with different micro/nanostructures are fitted by the following equation, ln(C_0_/C) = Kt. In this equation, C_0_ and C are the initial and real-time concentrations of the RhB solution. The results are demonstrated in Figure 6d. The slope of the fitted line shown in Figure 6d represents the photocatalytic performance of the sample. From the results demonstrated in this figure, it can also be seen that the photocatalytic activity of the obtained samples is according to the following order: rod-like Zn(OH)F < rod-like ZnO < hierarchical ZnO micro/nanostructures.

It is worth mentioning that the samples used in this work were all grown on zinc foils. Therefore, the obtained samples can be easily recycled and reused. The conventional powdery photocatalyst needs to be separated from the solution if it needs to be reused, which is disadvantageous for practical applications. In order to study the reusability and stability of the obtained photocatalysts, photocatalytic circulation experiments were carried out. The results corresponding to the three cycling runs of the photodegradation ability of the hierarchical ZnO micro/nanostructures are shown in Figure 6e. From these results, it can be seen that the photocatalytic performance of the prepared samples does not decline too much through the three cycling runs of the photodegradation of RhB. The results indicate that the obtained products are suitable for photocatalytic applications.

## 4. Conclusions

In summary, rod-like Zn(OH)F, rod-like ZnO, and hierarchical ZnO micro/nanostructures were prepared on zinc foils. The photocatalytic properties of the obtained micro/nanostructures were compared and studied. The hierarchical ZnO micro/nanostructures have better photocatalytic properties. The preparation of hierarchical ZnO micro/nanostructures increases the amount of ZnO loaded on the surface of the zinc foil. Therefore, the unique structural characteristics of hierarchical ZnO micro/nanostructure make it have a significantly enhanced photocatalytic performance. In addition, the hierarchical ZnO micro/nanostructures prepared on the surface of zinc foil also have the characteristics of reusability and stability.
